# Novel insights into the consequences of obesity: a phenotype-wide Mendelian randomization study

**DOI:** 10.1038/s41431-021-00978-8

**Published:** 2022-01-01

**Authors:** Chang He, Miaoran Zhang, Jiuling Li, Yiqing Wang, Lanlan Chen, Baiyu Qi, Jianping Wen, Jianli Yang, Sitong Lin, Dianyuan Liu, Ying Dong, Liying Wang, Qing Wang, Peng Chen

**Affiliations:** 1 Department of Genetics, College of Basic Medical Sciences, Jilin University, Changchun, 130000 China; 2 Department of Molecular Biology, College of Basic Medical Sciences, Jilin University, Changchun, 130000 China; 3 Department of Pathology, College of Basic Medical Sciences, Jilin University, Changchun, 130000 China; 4 School of Clinical Medicine, Jilin University, Changchun, 130000 China; 5 Department of Endocrinology, China-Japan Union Hospital, Jilin University, Changchun, 130000 China; 6 Department of Radiotherapy, The Tumor Hospital of Jilin Province, Changchun, 130000 China

**Keywords:** Lifestyle modification, Genetic predisposition to disease

## Abstract

Obesity is thought to significantly impact the quality of life. In this study, we sought to evaluate the health consequences of obesity on the risk of a broad spectrum of human diseases. The causal effects of exposing to obesity on health outcomes were inferred using Mendelian randomization (MR) analyses using a fixed effects inverse-variance weighted model. The instrumental variables were SNPs associated with obesity as measured by body mass index (BMI) reported by GIANT consortium. The spectrum of outcome consisted of the phenotypes from published GWAS and the UK Biobank. The MR-Egger intercept test was applied to estimate horizontal pleiotropic effects, along with Cochran’s *Q* test to assess heterogeneity among the causal effects of instrumental variables. Our MR results confirmed many putative disease risks due to obesity, such as diabetes, dyslipidemia, sleep disorder, gout, smoking behaviors, arthritis, myocardial infarction, and diabetes-related eye disease. The novel findings indicated that elevated red blood cell count was inferred as a mediator of BMI-induced type 2 diabetes in our bidirectional MR analysis. Intriguingly, the effects that higher BMI could decrease the risk of both skin and prostate cancers, reduce calorie intake, and increase the portion size warrant further studies. Our results shed light on a novel mechanism of the disease-causing roles of obesity.

## Introduction

Obesity is a global challenge that greatly impacts human health and behavior. Many studies have shown that obesity, generally defined as high body mass index (BMI), can lead to a range of physical and mental diseases including cardiovascular diseases (CVD) [1], type 2 diabetes (T2D) [2], depression [3], and cancers [4]. Some studies have indicated that blood traits, including high red blood cell (RBC), were associated with increased risk of obesity [5], while our understanding of the direct consequences of obesity remains unclear due to confounding factors that are not well controlled.

In the past few decades, observational studies have investigated the effects of high BMI on the risk of disease. Although diabetes was established to be the consequence of obesity [6], the effect of obesity on diabetic retinopathy (DR) is still blur [7, 8]. Furthermore, the association between BMI and blood iron status is strongly confounded by the age of the samples [9, 10].

Mendelian randomization (MR) is a powerful statistical approach that leverages genetic variants as instrumental variables (IVs) to investigate the causal effect of an exposure (e.g., obesity) on an outcome (e.g., CVD). Based on the random assignment of parental alleles to their offspring, a well-designed MR analysis is able to minimize the confounding effects and infer the causal effects as in a randomized clinical trial [11, 12].

In this study, we reported the results of a phenotype-wide MR analysis aimed at clarifying the direct causal effects of obesity on human health. To our knowledge, this is the first study to investigate the causal effects of high BMI on a broad spectrum of health outcomes. Our results not only validated the known consequences of obesity, but also identified novel obesity complications that deserve further attention in future studies.

## Materials and methods

### Ethics statement

The current study did not involve any identifiable personal information or intervention on the living subjects. Data sources of this study are publicly available. Therefore, this study was exempt from review by the institutional review board.

### Data collection and genome-wide association testing

Summary statistics of the BMI GWAS were obtained from Genetic Investigation of ANthropometric Traits (GIANT) Consortium (*N* = 339,224) [13]. The summary statistics of the UK Biobank (UKB) GWAS were calculated by Dr. Neale’s laboratory [14]. The summary statistics of the non-UKB GWAS were available from the MR-Base website [15]. The UKB is a large prospective cohort of above 500 thousand participants, who provided the responses to questionnaires and blood and urine samples at UKB recruitment centers [14]. Genomic data of ~820,000 variants were imputed up to a combined reference panel of 1000 Genome project and UK10K, resulting in 13.7 million genomic variants with imputation quality score ≥ 0.8 and MAF ≥ 0.0001. The GWAS summary statistics from Dr. Neale’s laboratory included ~300 thousand samples of European ancestry, aged between 40 and 69 years [16]. For each phenotype including BMI, the association test was conducted using a linear or logistic regression model adjusted for age, sex, square age, interaction of sex and age, interaction of sex and square age, and the first 20 PCs.

### Instrumental variables (IV) selection

The IVs should conform to the following MR principles: (1) significantly associated with the exposure (*p* value ≤ 5 × 10^−8^); (2) not associated with the outcome (*p* value > 0.05); (3) influence the outcomes only through the exposure. However, the second and the third principles could not be tested in practice. SNPs met above principles were pruned based on their pair-wise linkage disequilibrium (LD *r*^2^ < 0.01). The SNP of the smallest *p* value in a clump was selected as IV. The pleiotropy effect of IVs was estimated in our MR analysis as demonstrated in the following section.

In our primary MR analysis, there were 97 SNPs significantly associated with BMI (association *p* value ≤ 5 × 10^−8^) as reported by GIANT consortium [13]. After clumping, 92 SNPs were available for IV quality control. A SNP rs9641123 (hg19 chr7:g.93197732G>C) in *CALCR* gene was further removed because of being palindromic and allele frequency close to 50%. The association summary statistics of the 91 IVs is shown in Supplementary Table S1.

**Fig. 1:**
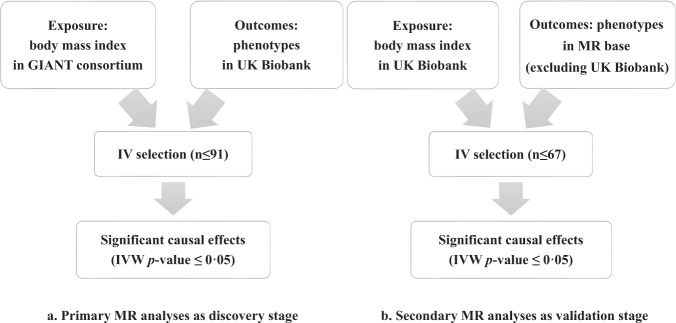
The workflow of our primary and secondary MR analyses. The flow diagram summarized the selection of the datasets and the instrumental variables in our primary MR analyses (**a**) and secondary MR analyses (**b**). GIANT the Genetic Investigation of ANthropometric Traits, IV instrumental variable, n numbers, MR Mendelian randomization, IVW inverse-variance weighted method.

In UKB data (Fig. 1), we got 51,998 significant BMI SNPs at genome-wide significant level of 5 × 10^−8^ with MAF ≥ 0.0001 and imputation quality score ≥0.8. After clumping (LD *r*^2^ < 0.01), 340 SNPs remained and were subjected to pleiotropic effect control in GWAS catalog. We further removed IVs that associated with a phenotype other than BMI or obesity at 5 × 10^−8^ to reduce the pleiotropic effects [17]. Finally, we employed 67 SNPs as IVs for our secondary MR analysis (Supplementary Table S2).

We calculated the *F*-statistic to evaluate the instrument strength of the IVs: *F* = ($\frac{{N - K - 1}}{K}$)($\frac{{R^2}}{{1 - R^2}}$), where *R*^2^ is the proportion of variance explained by IVs, *N* is the sample size from BMI GWAS, and *K* is the number of IVs.

### Mendelian randomization analysis

In our primary MR analyses, we employed BMI as the exposure and 4174 phenotypes in UKB data as the outcomes, including lifestyles, physical measurements, blood/urine assays, self-report, and diagnosed diseases. The association effects on BMI were estimated in the largest yet GWAS of BMI in GIANT consortium [13]. The secondary MR analysis, including 1077 outcomes from non-UKB GWAS from MR-Base, aimed to replicate the main findings in the primary analysis. The association effects of the measured BMI in UKB were estimated using 359,983 participants of European ancestry. In the MR analysis of RBC, the measured RBC (*N* = 350,475) in the UKB was the exposure, while the phenotypes from non-UKB GWAS were the outcomes.

Our two-sample MR analyses were all conducted following the practices recommended in the R package “TwoSampleMR” [15]. In brief, we first calculated the Wald ratio estimate for each IV and combined the estimates of all IVs by the inverse-variance weighted (IVW) method. The phenotype-wide IVW *p* values were corrected by false discovery rate (FDR) as proposed by Benjamini and Hochberg in 1995. We used MR-egger intercept and Cochran’s *Q* to estimate the pleiotropic effect and heterogeneity of the MR results, respectively. In the primary MR analyses, we report the significant causal effects with FDR ≤ 0.05, MR-egger intercept *p* value > 0.05, and Cochran’s *Q* *p* value > 0.05 [18]. In the secondary MR analyses, we sought validations at IVW *p* value ≤ 0.05. Furthermore, MR-PRESSO method was used to detect pleiotropy and bidirectional MR was performed for validated outcomes.

## Results

Our study design is shown schematically in Fig. 1. The *F*-statistics of IVs in the primary and secondary MR analyses are 76.55 and 23.82, respectively.

### The primary analyses results unveiled novel causal effects of obesity

The primary MR analysis investigated the causal effects of BMI on phenotypes reported in the UKB cohort.

**Fig. 2:**
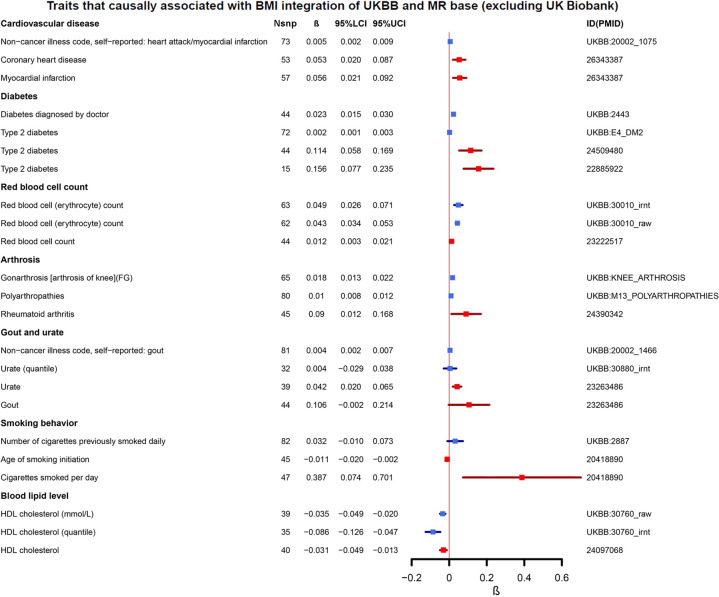
The forest plot of traits that causally associated with BMI integration of UK Biobank and MR-Base (excluding UK Biobank). N_SNP_ number of IVs, *β* the causal effect of IVW MR analysis, 95% LCI 95% lower confidence interval, 95% UCI 95% upper confidence interval, ID(PMID) the UK Biobank ID or the PubMed ID of the published GWAS. The primary analysis results from UKB are plotted in blue, while the secondary analyses result from UKB data are in red.

Overall, exposure to obesity could result in health problems in multiple systems (Fig. 2, Table 1, and Supplementary Table S3). First of all, higher BMI was associated with heart diseases (i.e., heart failure and myocardial infarction (MI)), diseases in the circulatory system (i.e., venous thromboembolism and blood clot), diabetes, hypertension, dyslipidemia, gout, and sleep disorders. Obesity patients were also prone to pain experience, probably caused by musculoskeletal problems (i.e., arthrosis in knee and hip). The health satisfaction and overall health rating were coded using higher scores indicating poorer health in the UKB data. Our results showed that higher BMI could contribute a poorer subjective health status. All these results were previously reported by other studies and further confirmed by us.

**Table 1:** Primary and secondary MR analyses results.

	Primary analyses (UKBB)				Secondary analyses (GWAS)						
Outcome	Beta	Pval	N_SNP_	Sample size (case/control)	Beta	Pval	N_SNP_	Sample size (case/control)	PMID	Global-Pval	Outlier-corrected
Diabetes diagnosed by doctor^a^	0.023	2.58 × 10^−9^	44	17,275/342,917	0.114	5.8 × 10^−5^	44	26,488/83,964	24509480	0.977	–
Urate	0.073	2.03 × 10^−5^	32	343,836	0.042	2.4 × 10^−4^	39	110,347	23263486	0.622	–
HDL cholesterol	−0.035	2.36 × 10^−6^	39	315,133	−0.031	7.5 × 10^−4^	40	187,167	24097068	0.670	–
Myocardial infarction	0.004	1.64 × 10^−2^	81	7018/354,176	0.056	2.0 × 10^−3^	57	43,676/128,199	26343387	0.590	–
Red blood cell count	0.043	1.66 × 10^−4^	63	350,475	0.012	7.9 × 10^−3^	44	66,214	23222517	0.823	–
Rheumatoid arthritis	0.003	3.50 × 10^−3^	87	4017/357,124	0.093	1.1 × 10^−2^	45	14,361/43,923	24390342	0.780	–
Age started smoking in former smokers	−0.047	3.91 × 10^−2^	82	88,898	−0.011	1.5 × 10^−2^	45	47,961	20418890	0.003	3.45 × 10^−6^
Number of cigarettes previously smoked daily	0.066	1.91 × 10^−3^	82	84,456	0.387	1.6 × 10^−2^	47	68,028	20418890	0.139	–
Gout	0.004	8.56 × 10^−4^	81	5174/355,967	0.106	5.4 × 10^−2^	44	69,374	23263486	0.087	–
Sleep disorders (combined)^b^	0.004	3.87 × 10^−4^	81	2951/358,243	−0.013	6.3 × 10^−2^	59	128,266	27494321	<5 × 10^−4^	5.04 × 10^−2^

Outcomes were the phenotypes reported in the UKB and GWAS.

*IVW_beta and IVW_pval* the effect size and *p* value of IVW MR analysis, *N*_*SNP*_ the number of IVs in the analysis, *HDL cholesterol* high-density lipoprotein cholesterol, *global-Pval* the pval of MR-PRESSO global test to detect horizontal pleiotropic effect, *Outlier-corrected* corrected MR results with outliers being removed to correct horizontal pleiotropic effect.

^a^The phenotype of interest was type 2 diabetes in secondary analyses.

^b^The phenotype of interest was sleep duration in secondary analyses.

**Fig. 3:**
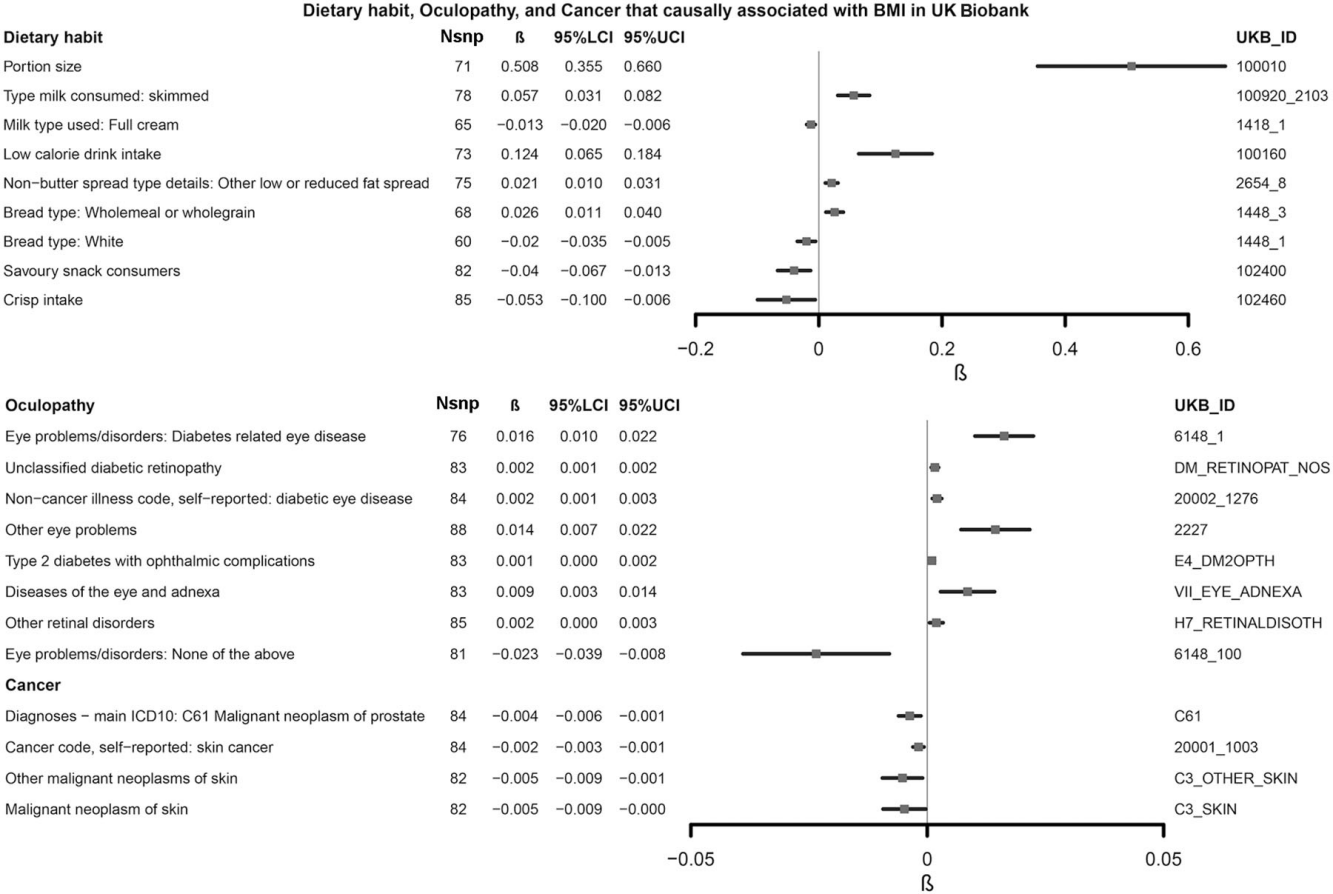
The forest plot of the causal effects of obesity on diet preferences, oculopathy, and cancer UK Biobank. N_SNP_ number of IVs, *β* the causal effect of IVW MR analysis, 95% LCI 95% lower confidence interval, 95% UCI 95% upper confidence interval, UKB_ID the UK Biobank ID.

Our novel results implicated that obesity could increase the RBC in peripheral blood. One SD increase of BMI could result in the elevation of 0.043 × 10^12^/L RBC [95% CI: 0.034–0.063, unit: 10^12^/L]. Intriguingly, our results suggested that higher BMI could increase the risk of unclassified DR (IVW beta = 0.002 [0.001, 0.002], IVW *p* value = 4.18 × 10^−5^). At the same time, obesity decreased the risks of prostate cancer and skin cancer (Fig. 3). However, the causal effects on other types of cancers, including lung cancer, stomach cancer, and leukemia, were not significant (IVW *p* value > 0.05). It should be pointed out that the portion size of obese people increased significantly (Fig. 3). Meanwhile, we observed a clear trend that obesity led to the diet preference on low fat and high fiber foods. This trend was evidenced by the increased preference on skimmed milk over half-skimmed or full cream milk and wholegrain over white bread, along with increased BMI.

### Secondary MR validated a proportion of our primary findings

We were able to validate the causal effects of BMI on diabetes, MI, arthritis, dyslipidemia, cigarettes smoked per day, and RBC (Fig. 2 and Table 1). Therein, RBC is the only validated novel finding. In addition, the effects on sleep duration (IVW *p* value = 0.063, IVW *β* = −0.013) and gout (IVW *p* value = 0.054, IVW *β* = 0.106) were in the same direction as in the primary analyses, although at a marginal significance level (Supplementary Table S4). Moreover, obesity could increase the serum urate level, with 1 SD increase of BMI results in 4% more urate. Due to the absence of genome-wide summary statistics, diabetes-related eye diseases, dietary habit traits, prostate, and skin cancers were not validated in our secondary analysis.

### RBC MR analyses result

To reveal the consequences of abnormal RBC, we estimated the causal effect of RBC using the approach consistent to our secondary MR analysis. This MR analysis was conducted with RBC in UKB data as exposure and phenotypes in non-UKB GWAS as outcomes. We finally got 438 IVs for the RBC MR analyses after LD-based pruning (Supplementary Table S5).

In this stage, we found that higher RBC could cause higher 2-h blood glucose level during oral glucose tolerance test (IVW *β* = 0.22, 95% CI: 0.06–0.37, IVW *p* value = 0.007) and increase the risk of T2D (IVW *β* = 1.51, 95% CI: 0.23–2.69, IVW *p* value = 0.013).

### Bidirectional Mendelian randomization analyses results

We treated RBC, smoking behavior, diabetes, urate, MI, HDL cholesterol, rheumatoid arthritis, portion size, and low calorie as exposures, but found no significant causal association with BMI (Supplementary Table S6).

## Discussion

Studying the causal relationship between obesity and various human diseases can help guide the decisions in health management and disease prevention strategy. Our study validated known clinically relevant obesity complications and uncovered novel causal effects of high BMI. Based on our MR analyses integrated with UKB data and published GWAS, we confirmed many consequences that have been previously reported by observational and experimental studies (Fig. 2), such as CVD, T2D, and dyslipidemia [1, 19–21]. As for the reverse causation between BMI and T2D, the results vary among observational studies where the difference in the courses of T2D usually leads to variation in nutrition and weight loss [22, 23]. In addition, it should be noted that the European Association for the Study of Diabetes and European Society of Cardiology guideline recommend T2D patients to control weight, hoping to reduces potential risk factors [24]. Furthermore, our results further support the known causal links between obesity and urate concentration, arthritis, and smoking behavior that have been reported by previous MR studies [25–29]. As for novel finding, we found that higher BMI could increase the RBC and proportion of low-calorie foods, and further lead to bigger portion size. Therein, the causal relationship between BMI and RBC was validated in the replication stage.

According to our both MR analyses, the results consistently demonstrated that genetically elevated BMI was associated with increased RBC. This phenomenon could be attributed to hypoxia in obese subjects. Compared with non-obese subjects, obese subjects displayed significantly lower adipose tissue blood flow and muscle blood flow rates [30]. A previous study indicated that PO_2_ (partial pressure of oxygen) levels are lower in obese subjects than in lean subjects, using electrode-based O_2_ measurement [31]. Overall, these studies strongly suggest that hypoxia is a consequence of obesity. Human body has evolved a series of mechanisms to balance the stress induced by hypoxia including increasing RBC [32]. RBCs release ATP, which can stimulate vasodilatation and increase blood flow in response to deoxygenation [33]. Previous studies have suggested that RBCs are associated with metabolic syndrome, insulin resistance, and fatty liver disease [34]. In our MR analysis of RBC, we found a causal association between elevated RBC and the risk of T2D. Given the causal effects of obesity-RBC and obesity-T2D, this result suggested that elevated RBC may be a mediator in the pathogenesis of obesity-induced T2D, although the mechanism may be more complicated than we have observed.

Apart from the novel finding of RBC, we also identified that higher BMI could lead to an increased risk of MI, which was in line with previous epidemiological studies (Fig. 2 and Table 1). It is widely believed that obesity increases the risk of CVD [1]. However, whether BMI is a direct risk factor for MI is still under debate [35, 36]. Several studies have suggested that higher BMI is strongly associated with the risk of MI [35, 37]. They are supported by our MR analysis with a significant genetic link between obesity and MI. These results may imply that promote weight loss may be beneficial in lowering the risk of MI.

Because of the absence of certain UKB outcomes from GWAS catalog and the difference in phenotype definitions between the two, not all findings in our primary MR analysis were subjected to the secondary MR analysis. DR is the most common ophthalmic complication of diabetes and is a leading cause of acquired blindness [38]. In recent years, various clinical epidemiological studies have investigated the effects of high BMI on the risk of DR. Some studies showed that people who are overweight or obese are less vulnerable to DR [39, 40], while other studies did not detect a significant association between obesity and DR [41, 42]. Thus, the potential genetic links between obesity and DR require further investigation. We demonstrated that high BMI was not only a major risk factor for T2D that serves as a major cause of retinopathy, but also an independent causal factor for ophthalmic diseases. This is helpful in detangling the complicated relationship between obesity and DR. In fact, the relationship between BMI and DR is complicated by the course of T2D, which could induce weight loss [22]. As a result, lower BMI in T2D patients is more likely to be a concomitant symptom of DR after a prolonged course of T2D, rather than a causal factor of DR. Therefore, observational studies could be confounded by this U-shaped curve between BMI and DR incidence [43]. We think stratified analysis or sliding window analysis could be informative when the individual-level data are available. However, our MR results could be biased by the hidden pleiotropic effect from T2D of the IVs. The complex causal relationship between BMI and DR has to be further studied.

Obesity is associated with the susceptibility to various cancers [44]. In our study, a higher BMI reduced the risk of skin cancer and prostate cancer (Fig. 3). The inverse correlation between obesity and skin cancer incidence rate has been observed in a US Caucasian cohort [45]. The authors concluded that obesity may be the mediator between chronic sun exposure and skin cancer. However, we observed a direct effect in our study, which indicated that genetic-driven obesity may be protective against the aberrant changes that result from sunlight exposure. Moreover, a large body of literature has established the complex relationship between obesity and prostate cancer, while the effect of obesity on specific stages of prostate cancer varied. People with higher BMI were less likely to have early-stage prostate cancer but were susceptible to aggressive cancer [46–48]. In our study, the MR analysis of obesity and prostate cancer was not stratified by prostate cancer stages. However, the protective effect was robust, in that the causal effect was consistent across our primary and secondary analysis.

Our study also uncovered a relationship between obesity and dietary preferences (Fig. 3), including an increase in the proportion of low-calorie foods and bigger portion size. The former looks straightforward, owing to the widespread of health education, encouraging obese people to consume less calorie to manage weight, while the latter can be more complex because of the interaction of physiological and psychological factors associated with food consumption. For instance, leptin resistance has been reported to make it relatively harder for obese people to sense fullness [49]; the awareness of already taking a low-calorie diet tended to relax the vigilance about the total amount of food [50]. Meanwhile, recent studies indicated a significant association between obesity and high BMI with eating disorder [51–53]. Zoe et al. suggested that those with higher BMI were more likely to suffer from binge eating or overeating [51]. Similarly, people on dietary restraint usually fell into the illusion that they should take more snacks to compensate for relatively low consumption, making their actual portion size much bigger [54, 55]. The reversal analysis implicated that the intake of low-calorie foods or the use of a large portion size did not adequately lead to obesity. A possible explanation might be that the food structure and the amount of food could not determine the onset of obesity alone in the context of modern lifestyles since obesity is a disease determined by multiple factors. Thus, more investigations focused on comprehensive indices should be conducted in the future.

## Conclusion

**Fig. 4:**
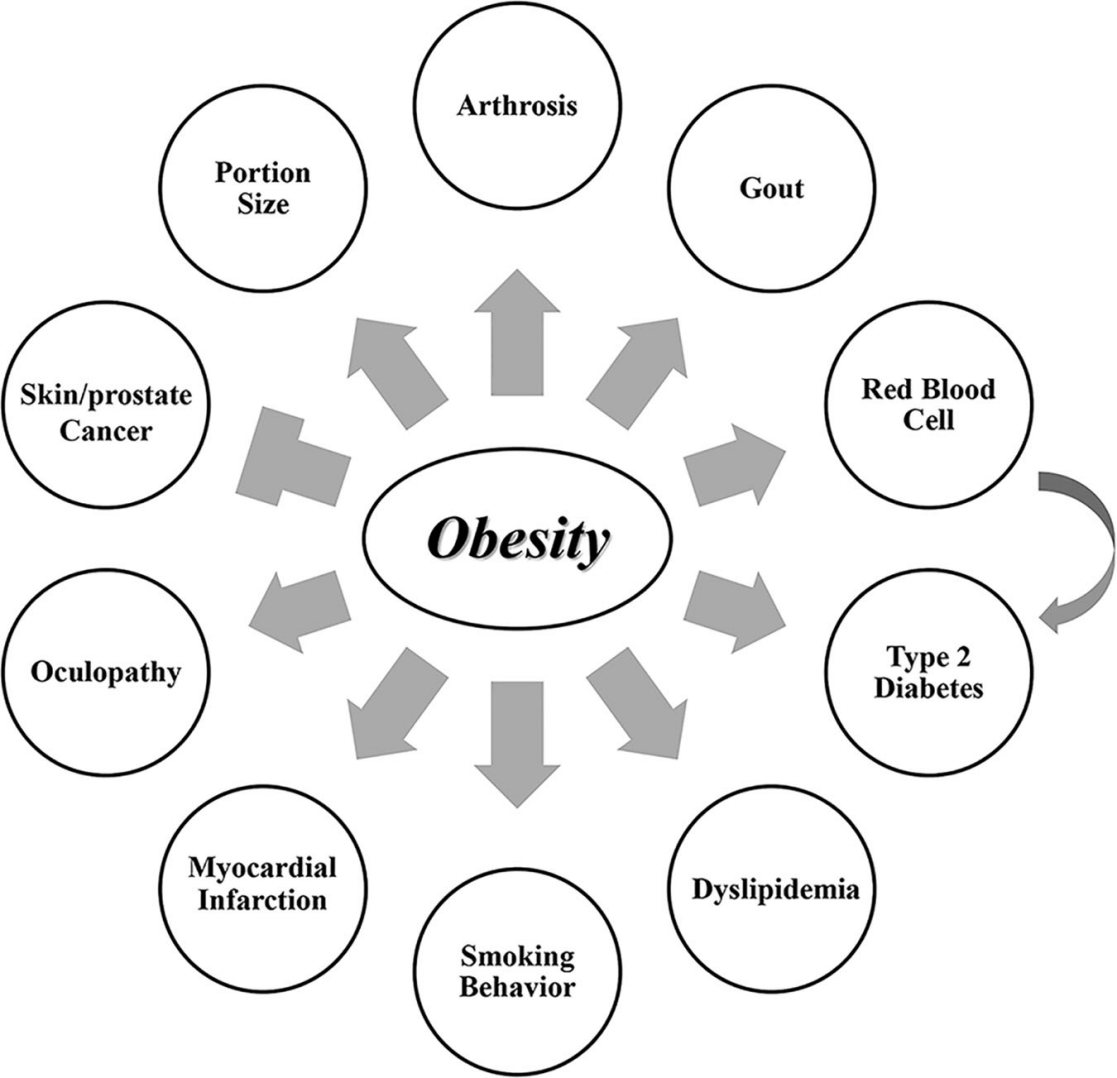
The illustrative chart of the consequences of obesity. Obesity increased the risk of an outcome or the level of a quantitative trait when they are connected by sharp arrow. On the contrary, obesity decreased the risk of the outcome when they are connected by blunt arrow.

Our comprehensive MR study robustly demonstrated that obesity is causal of a variety of human diseases (Fig. 4). Our results confirmed the known causal effect of obesity on human health. Most importantly, our study discovered novel consequences (e.g., MI and RBC) and further supported several consequences under debate (e.g., DR, skin cancer, and prostate cancer). Our results could be implicative to disease prevention and future research works on disease etiology.

### URLs

UK Biobank, https://www.ukbiobank.ac.uk/

Dr Neale’s Lab, http://www.nealelab.is

GIANT consortium, http://portals.broadinstitute.org/collaboration/giant/index.php

GWAS catalog, https://www.ebi.ac.uk/gwas

## Supplementary information

Supplementary table legends

Table S1

Table S2

Table S3

Table S4

Table S5

Table S6

## Data Availability

The datasets analyzed during the current study are available in the Dropbox cloud storage provided by the Neale Lab (https://www.nealelab.is/uk-biobank, round 2 GWAS) and the webapp of MR-Base (http://app.mrbase.org).
