# Supplementary material for: Novel insights into the consequences of obesity: a phenotype-wide Mendelian randomization study
Source: Eur J Hum Genet. 2022 Jan 1;30(5):540–6. doi: 10.1038/s41431-021-00978-8 (PMC9091238; doi:10.1038/s41431-021-00978-8)
Supplement: Supplementary file 1 — Supplementary table legends [file 41431_2021_978_MOESM1_ESM.docx]

**Supplemental Data** (Supplemental Data include six tables)

**Table S1.** Instrumental variables in primary analyses.

Abbreviations: SNP, single-nucleotide polymorphism; Gene was the nearby gene of SNP; EA, effect allele/alternative allele; OA, other allele/reference allele; Beta, standard deviation change in telomere length per copy of the effect allele; SE, standard error; EAF, effect allele frequency; Chr, chromosome; Pos, base-pair position (GRCh37) ; *, the overlap (LD proxy r^2^≥0.3) IV in primary and secondary MR analyses.

**Table S2.** Primary MR analyses result in UK biobank.

Abbreviations: Outcomes were the phenotypes in UK biobank. Outcomes were the phenotypes. Nsnp, number of IVs; Beta, SE, *p* Value, and FDR_Pval, the causal effect, the standard error, the p-value, and the p-value corrected by false discovery rate of IVW MR analysis; Q_pval, the p-value of Cochran’s Q value; Egger _pval, the p-value of MR-Egger intercept.

**Table S3.** Instrumental variables in secondary MR analyses.

Abbreviations: SNP, single-nucleotide polymorphism; Gene was the nearby gene of SNP; EA, effect allele/alternative allele; OA, other allele/reference allele; Beta, standard deviation change in telomere length per copy of the effect allele; SE, standard error; EAF, effect allele frequency; Chr, chromosome; Pos, base-pair position (GRCh37) ; *, the overlap (LD proxy r^2^≥0.3) IV in primary and secondary MR analyses.

**Table S4.** Secondary MR analyses result in MR base (excluding UK biobank).

Abbreviations: MR-base_ID and PMID are the ID in MR-base and PubMed database. They are used to refer to the study which reported the GWAS on the outcomes. Nsnp, number of IVs; Beta, Se, and *p* Value, the causal effect, the standard error and the p-value of causal effect of IVW MR analysis; Q_pval, the p-value of Cochran’s Q value; Egger_pval, the p-value of MR-Egger intercept.

**Table S5.** Instrumental variables of RBC count.

Abbreviations: SNP, single-nucleotide polymorphism; Gene was the nearby gene of SNP; EA, effect allele/alternative allele; OA, other allele/reference allele; EAF, effect allele frequency; Chr, chromosome; Pos, base-pair position (GRCh37); Beta, standard deviation change in telomere length per copy of the effect allele; SE, standard error.

**Table S6.** Bi-directional Mendelian randomization analysis results.

Abbreviations: Exposure_ID, the ID in MR-base. Nsnp, number of IVs; Beta, Se, and *p* Value, the causal effect, the standard error and the p-value of causal effect of IVW MR analysis.
